# Optimum water depth ranges of dominant submersed macrophytes in a natural freshwater lake

**DOI:** 10.1371/journal.pone.0193176

**Published:** 2018-03-07

**Authors:** Bibi Ye, Zhaosheng Chu, Aiping Wu, Zeying Hou, Shengrui Wang

**Affiliations:** 1 State Key Laboratory of Environmental Criteria and Risk Assessment, State Environmental Protection Key Laboratory for Lake Pollution Control, Research Center for Lake Ecology and Environments, Chinese Research Academy of Environmental Sciences (CRAES), Beijing, China; 2 Ecology Department, College of Bioscience & Biotechnology, Hunan Provincial Key Laboratory of Rural Ecosystem Health in Dongting Lake Area, Hunan Agricultural University, Changsha, China; Shandong University, CHINA

## Abstract

Macrophytes show a zonal distribution along the lake littoral zone because of their specific preferred water depths while the optimum growth water depths of dominant submersed macrophytes in natural lakes are not well known. We studied the seasonal biomass and frequency patterns of dominant and companion submersed macrophytes along the water depth gradient in Lake Erhai in 2013. The results showed that the species richness and community biomass showed hump-back shaped patterns along the water depth gradient both in polydominant and monodominant communities. Biomass percentage of *Potamogenton maackianus* showed a hump-back pattern while biomass percentages of *Ceratophyllum demersum* and *Vallisneria natans* appeared U-shaped patterns across the water depth gradient in polydominant communities whereas biomass percentage of *V*. *natans* increased with the water depth in monodominant communities. Dominant species demonstrated a broader distribution range of water depth than companion species. Frequency and biomass of companion species declined drastically with the water depth whereas those of dominant species showed non-linear patterns across the water depth gradient. Namely, along the water depth gradient, biomass of *P*. *maackianus* and *V*. *natans* showed hump-back patterns and biomasses of *C*. *demersum* displayed a U-shaped pattern in the polydominant communities but biomass of *V*. *natans* demonstrated a hump-back pattern in the monodominant communities; frequency of *P*. *maackianus* showed a hump-back pattern and *C*. *demersum* and *V*. *natans* maintained high frequencies in the two types of communities. We can speculate that in Lake Erhai the optimum growth water depths of *P*. *maackianus* and *C*. *demersum* in the polydominant communities are 2.5–4.5 m and 1–2 m or 5–6 m, respectively and that of *V*. *natans* is 3–5 m in the polydominant communities and 2.5–5 m in the monodominant communities. This is the first report that the optimum water depth ranges in the horizontal direction of three dominant submersed macrophytes in a natural freshwater lake were determined.

## Introduction

Observational and experimental studies demonstrate that macrophytes distribute zonally along lake littoral zones because of their specific preferred water depths [1], and their distribution is determined by numerous factors [2–5]. Among these factors, water depth is one of the most important factors that affect the distribution of macrophytes [6–7], which are greatly sensitive to water level fluctuation [7–8]. Submersed species adapt to different water levels by their special morphological and physiological adjustments, resulting in submersed plants distributing in different water depth ranges [9]. For example, *Ceratophyllum demersum* inhabits a broad water depth range whereas *Potamogeton lucens* grows at a limited water depth range [7, 10]. For submersed plants, light is sufficient but competition, disturbance and shading may be great in the shallow water whereas strong competition, disturbance and shading can be avoided but low light stress is unavoidable in the deep water [11–12]. Consequently, submersed species have to select their own optimum water depth ranges to grow and propagate by trade offing these advantages and disadvantages. For purpose of restoring submersed macrophytes, optimum water depths of many submersed plants have been established in many controlled field and laboratory experiments, which are usually short of competition from other submersed plants because of limited study species or where the optimum water depths are analyzed only in a vertical direction [7, 9, 13–15]. Actually, submersed aquatic plants have to compete for light with emergent, floating and other submersed macrophytes in natural environments [11]. Furthermore, water depth stratification of submersed species occurs in both vertical and horizontal directions due to habitat filtering and niche differentiation [1, 7, 9]. However, very few studies focus on this stratification in the horizontal direction [7, 9]. Accordingly, the optimum water depth rang of a given submersed species, especially in the natural water bodies, is far from clear and we can not speculate it from its morphological and physiological responses to water depth as stated in most previous works [7, 9, 13–15].

Presence/absence study is one of the good methods studying the environmental regulation of plant distribution because it can quantitatively define the environmental limits of a give species [16]. Similarly, biomass measurement discriminates the quantitative differences in plant abundance easily as a plant’s biomass is sensitive to its growth [16]. Therefore, biomass sampling is another good method studying the environmental regulation of plant distribution. In this study, we chose frequency and biomass to study the optimum water depth range for a given submersed species in a natural freshwater lake with relatively high species richness.

In natural communities, one or more species can dominate in one communities. The former is a momodominant community and the latter is a polydominant community. Species distribution and assembly would be different between monodominant and polydominant communities since dominant species exert significant effects on the species richness [17]. For example, some dominant macrophytes out-compete other species for light by rapidly producing above-ground photosynthetic tissues [17]. As far as submersed species are concerned, canopy former and erect species, typically shade-intolerant, prefer the shallow water while bottom dweller and rosette species, usually shade-tolerant, dwell in the deep water to avoid strong competition [14, 18]. We can speculate that species distribution and assembly would be different between shade-intolerant species and shade-tolerant species across the water depth gradient.

In this study, we chose Lake Erhai as our study lake because it is rich in submersed macrophytes although submersed plants are declining in recent years due to the rapid economy and population growth [19–20]. The aim of the project was to determine the optimum water depth ranges of the dominant aquatic species by comparing the frequency and biomass of the submersed species along the water depth gradient in Lake Erhai. Before analyzing, we categorized the study communities into monodominant and polydominant communities due to their significantly different numbers of dominant submersed macrophytes.

## Material and methods

### Study area

**Fig 1:**
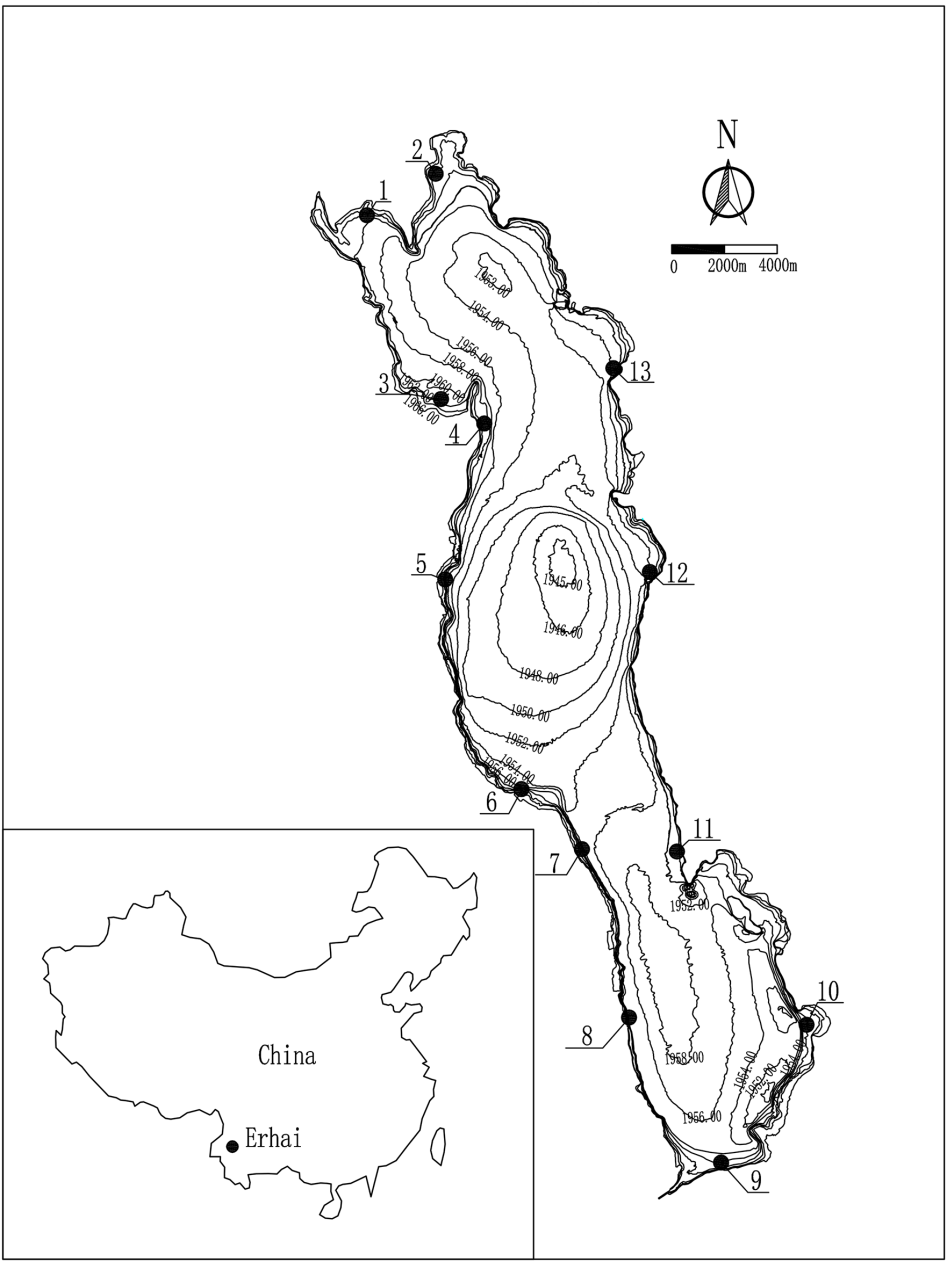
The thirteen studied transects in Lake Erhai, S1-S10 dominated by *Ceratophyllum demersum*, *Potamogeton maackianus* and *Vallisneria natans*; S11-S13 dominated by *V*. *natans*.

Lake Erhai (25°36’-25°58’ N, 100°06’-100°18’ E) is located in Yunnan Province, Southwest China. It is a plateau lake and is 42.58 km long and 8.0 km wide with a 251.0 km^2^ water surface area and a 2,656 km^2^ watershed. The average depth and maximum depth of Lake Erhai are 10.8 m and 21.3 m, respectively, corresponding to a 2.96 × 10^9^ m^3^ water volume (Fig 1). This lake is located in a warm plateau climate and the monsoon results in an annual precipitation of 1024 mm, primarily in summer. The mean annual temperature, sunshine duration and frost-free period are 15.6°C, 2345 hours and 228 days, respectively.

### Field sampling

In Lake Erhai, we selected thirteen (1–10 in the gentle slope area and 11–13 in the steep slope area) transects (excluding recent human activities as mowing and fishing) to study the distribution pattern of submersed macrophytes (Fig 1), and the length of each transect was about 60–100 m. At each transect, six 0.2 m^2^ plots were haphazardly surveyed along an offshore distance gradient at 3 m and 1 m intervals horizontally parallel to the water surface in a boat in the gentle slope area and steep slope area, respectively, until the water depth reached 7.0 m depth, which is the maximum water depth macrophytes distributed [21]. In each plot, all the species were identified and recorded in March (Spring), June (Summer), September (Autumn) and December (Winter) in 2013. The recorded water levels of the four seasons were 1973.62, 1973.18, 1974.16 and 1974.08 meters above the mean sea level, respectively. We used a rotatable reaping hook (diameter = 0.5 m, area = 0.2 m^2^) to sample macrophytes in a boat. Almost all macrophytes within the plot were uprooted and the biomass of each species was weighed after rinsing off all sediment and removing the excess water. Furthermore, qualitative measurement of the shallowest and deepest water depth of each submersed plant was also conducted simultaneously. We state clearly that no specific permissions were required and the field studies did not involve any endangered or protected species. Moreover, no vertebrates were studed in this research.

We sorted the macrophyte species into three categories based on the biomass percentage of an individual species in a transect community: dominant species with a biomass percentage more than 20%, companion species with a biomass percentage between 1% and 20%, and rare species with a biomass percentage below 1%. Based on the number of dominant species, we divided the thirteen transect communities into two types: polydominant community (S1-S10) and monodominant community (S11-S13). In the polydominant community, *Ceratophyllum demersum*, *Potamogeton maackianus* and *Vallisneria natans* were the dominant species; *Myriophyllum spicatum*, *Hydrilla verticillata*, *Potamogenton lucens*, and *Potamogeton malaianus* were the companion species and the other seven species were rare species. In the monodominant community, *V*. *natans* was the dominant species; *C*. *demersum*, *P*. *maackianus* and *M*. *spicatum*, *H*. *verticillata* were the companion species and the other eight species are rare species. Due to the limited data of rare species, only dominant and companion species were analyzed in this study. Species and community biomasses in each water depth were calculated as the average biomass of each species and the total species, respectively.

For analysis, we set every 50 cm range of water depth as a water depth, such as water depths from 1 cm to 50 cm were considered as the water depth of 50 cm and water depths from 51 cm to 100 cm were considered as the water depth of 100 cm and so on. Seasonal biomass of each species at each water depth was Ln-transformed to make the species biomass distribution more visible along the water depth gradient. To analyze the competitiveness of the dominant species at each water depth, the biomass percentage of each dominant species at each water depth was calculated as the biomass of the individual species divided by the total biomass. Similarly, the frequency of each species at each water depth was calculated as the number of times the species present in the plots divided by the total number of sample plots. The distribution range of water depth of each species was calculated as the maximum water depth of the individual species subtracted the minimum water depth of the individual species after standardizing all values as the summer water level.

### Statistical analysis

A two-sample Welch’s t test was used to determine the differences of the minimum, maximum and ranges of water depth of individual species between polydominant and monodominant communities. The differences among biomass percentages of the three dominant species in the polydominant communities were tested by analysis of variance (ANOVA) using Tukey’s Honest Significant Difference test. All ANOVA was measured at 95% confidence level (SPSS 17.0, SPSS Inc., Chicago) and homogeneity of variances was tested by Levene’s test. The independence and normality were tested based on the following model:

$$Y_{i,j}\;=\;\mu +\alpha_{i}+\epsilon_{i,j}$$

where Y_i, j_ is the j-th observation of the response variable in group i, μ is the overall mean, α_i_ is the effect of group i, and ϵ_i, j_ is the random statistical error in observation Y_i, j_, assumed to be independent and normally distributed with mean 0 and variance σ^2^ > 0 for all i and j.

Relationships between community biomass and species richness across the water depth gradient were determined by the following binary linear regression model after standardizing all water depths as the winter water level:

$$Y_{j}\;=\;\beta_{0}+\beta_{1}x_{i}+\beta_{2}x_{j}^{2}+\xi_{j}$$

where Y_j_ is the j-th observation of the response variable, x_j_ is the j-th observed value of the predictor variable (depth), the β_j_ are the regression coefficients, and ξ_j_ is the random statistical error in observation Y_j_, assumed to be independent and normally distributed with mean 0 and variance σ^2^ > 0 for all j. A potential problem of our statistics is that our sampling plots maybe spatial auto-correlated because our data are from different plots in the same sampling transect.

## Results

### Responses of biomass and species richness

**Fig 2:**
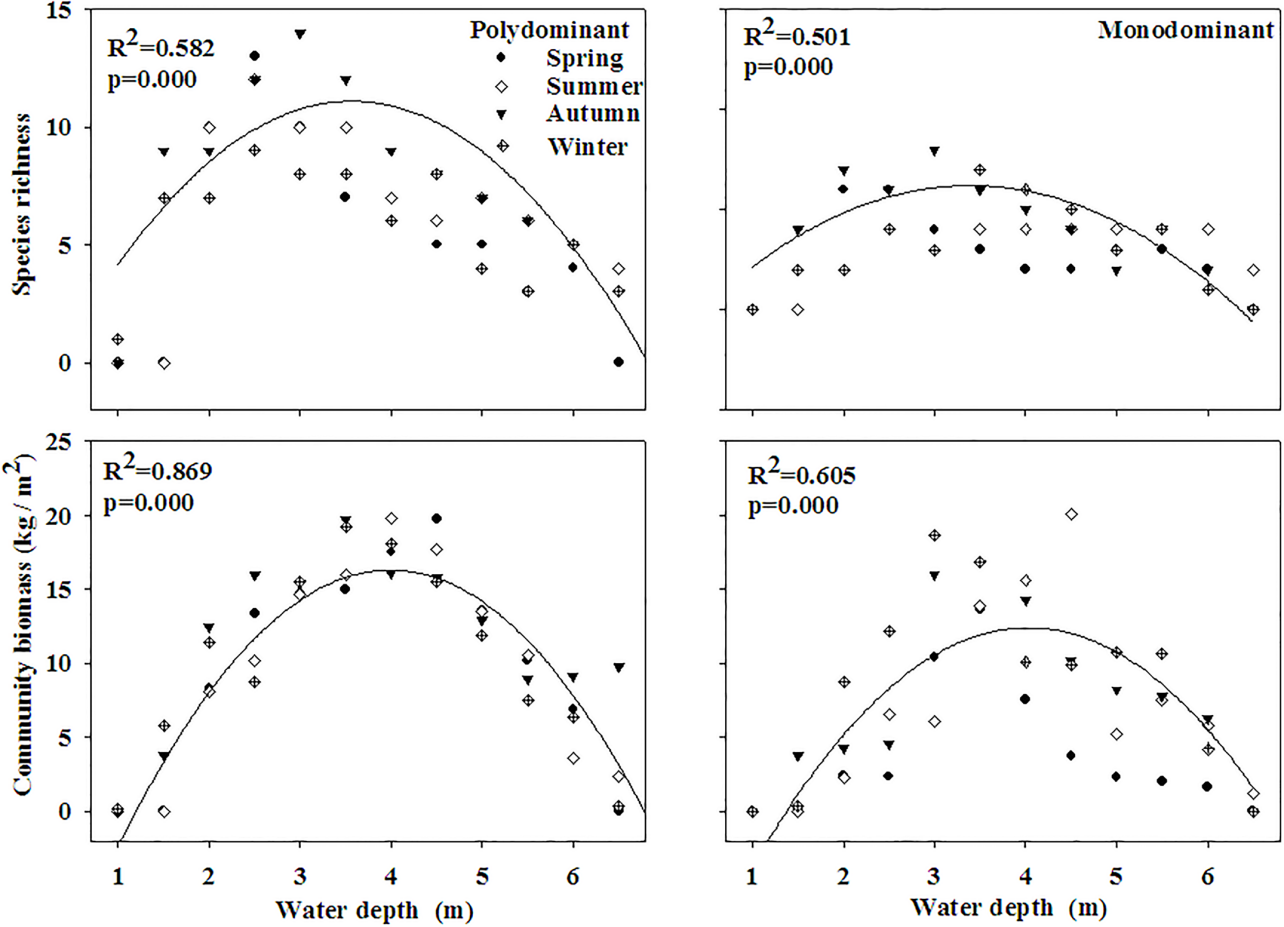
Relations between water depth and species richness, community biomass in the polydominant and monodominant communities.

**Fig 3:**
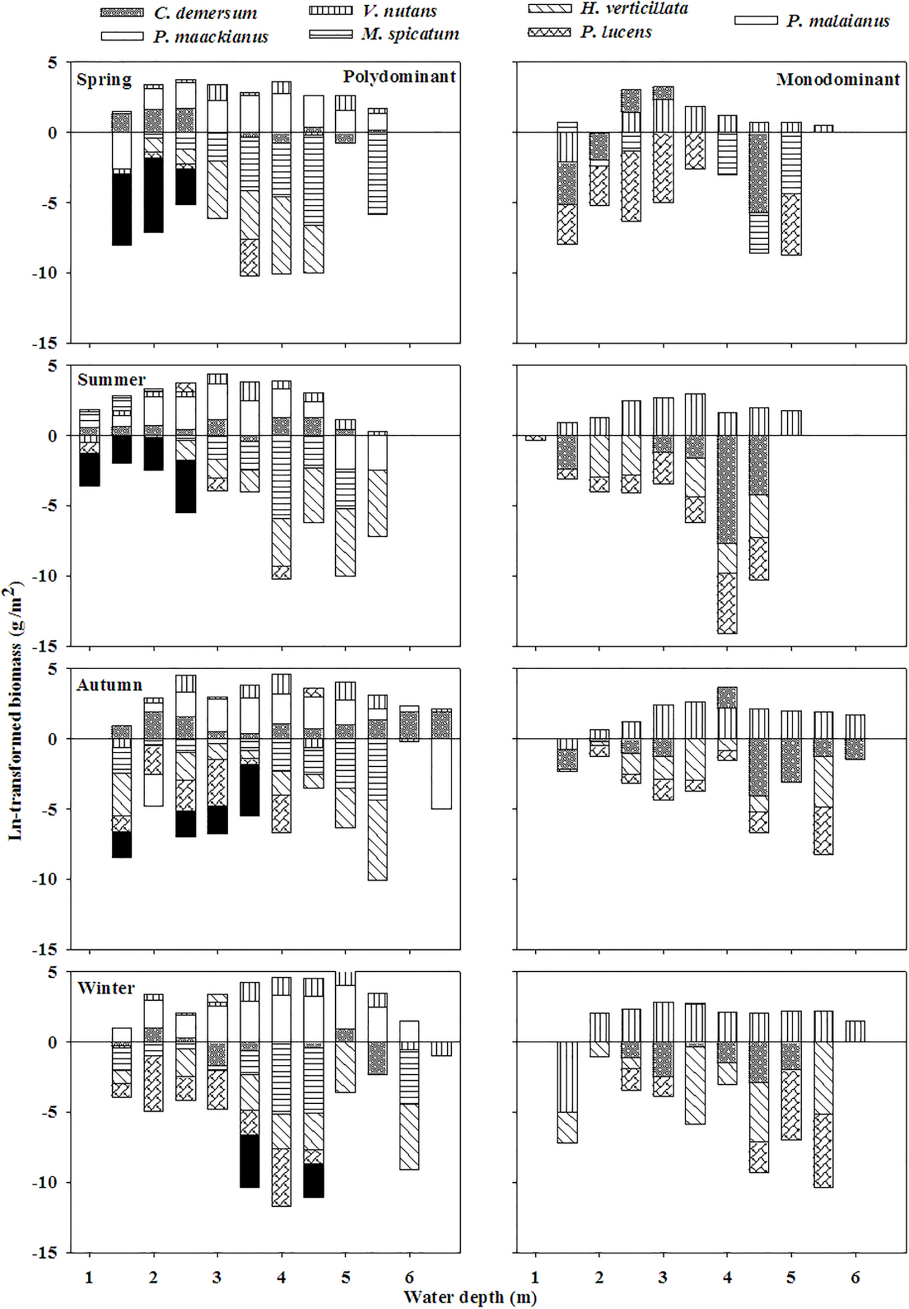
Seasonal biomass patterns of submersed species across the water depth gradient in the polydominant and monodominant communities.

A total of eighteen submersed species (attached to seven families and nine genera) were recorded in our survey (S1 Table): Potamogetonaceae (10), Hydrocharitaceae (3), Najadaceae (1), Ceratophyllaceae (1), Haloragaceae (1), Lentibulariaceae (1), Characeae (1). The eighteen submersed macrophytes were all recorded in the polydominant communities and only thirteen submersed macrophytes were recorded in the monodominant communities. Community biomass and species richness of both polydominant and monodominant communities showed hump-back relationships with the water depth (Fig 2). In polydominant communities, biomass of *P*. *maackianus* and *V*. *natans* demonstrated hump-back response patterns across water depth gradient whereas biomass of *C*. *demersum* demonstrated a U-shaped response patterns and biomasses of the other species demonstrated linear pattern along the gradient (Fig 3). In monodominant communities, biomass of *V*. *natans* and *C*. *demersum* displayed hump-back response patterns across the water depth gradient whereas biomasses of the other species displayed linear pattern along the gradient (Fig 3).

**Fig 4:**
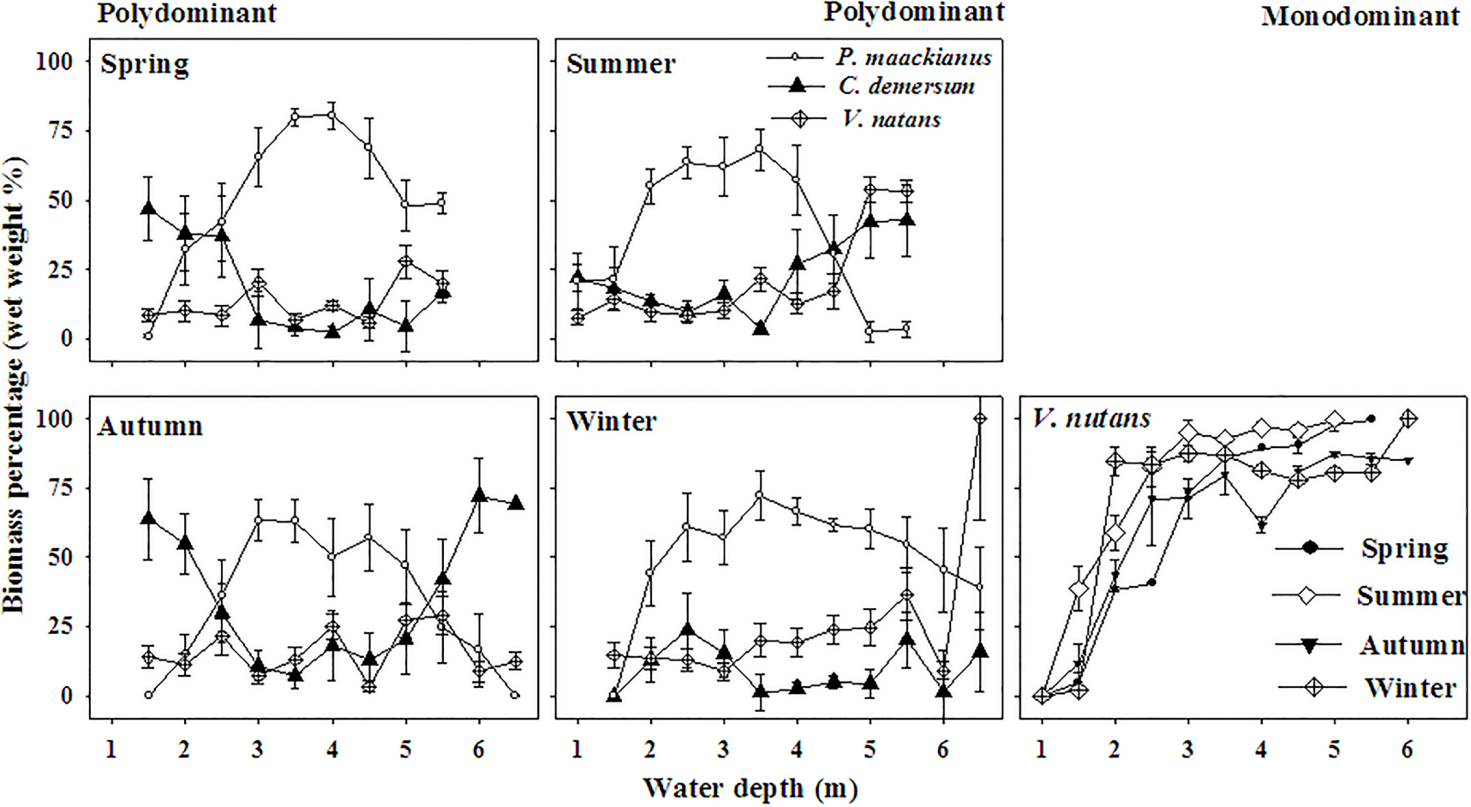
Seasonal biomass percentages of the dominant species across the water depth gradient in the polydominant and monodominant communities.

Generally, biomass percentage of the dominant *P*. *maackianus* exhibited a hump-back response pattern across the water depth gradient whereas biomass percentage of *C*. *demersum* and *V*. *natans* exhibited U-shaped response patterns along the gradient in the polydominant communities (Fig 4). Furthermore, biomass percentage of the dominant *P*. *maackianus* was higher [F _(2, 141)_ = 13.303, p = 0.000] than that of *C*. *demersum* and *V*. *natans* (Fig 4). However, biomass percentage of the dominant *V*. *natans* increased drastically with the increase of water depth in the four seasons in the monodominant communities (Fig 4).

### Minimum, maximum and range of water depth

**Fig 5:**
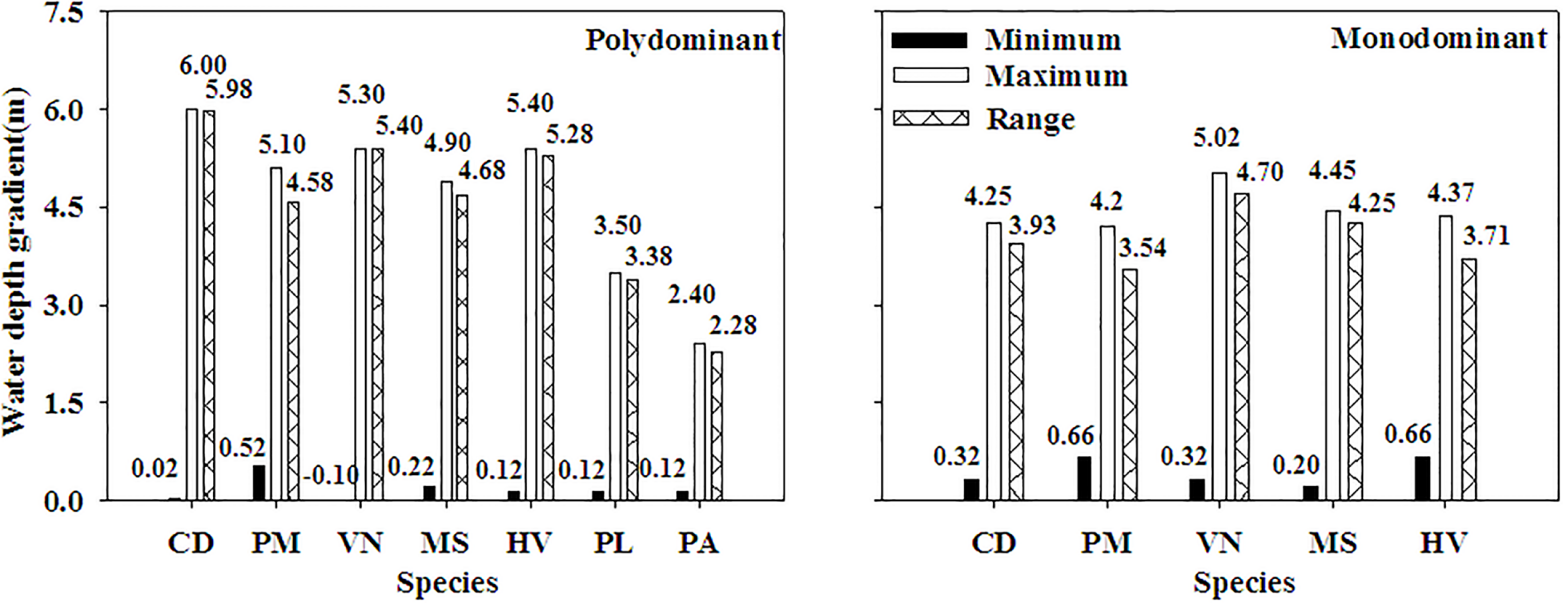
The minimum, maximum and range of water depth of submersed macrophytes in the polydominant and monodominant communities. CD: *Ceratophyllum demersum*; PM: *Potamogeton maackianus*; VN: *Vallisneria natans*, MS: *Myriophyllum spicatum*; HV: *Hydrilla verticillata*; PL: *Potamogeton lucens*; PA: *Potamogeton malaianus*; values shown on the top of each bar.

In general, the maximum, minimum and range of water depth of *P*. *lucens* and *P*. *malaianus* were lower, higher (except *C*. *demersum* and *M*. *spicatum*) and narrower than those of the other species and the lowest minimum, highest maximum and broadest range of water depth occurred on *V*. *natans*, *C*. *demersum* and *C*. *demersum*, respectively, in the polydominant communities (Fig 5). However, *V*. *natans* grew in higher maximum, lower minimum (except *M*. *spicatum*) and broader range of water depth than the other species and the lowest minimum, highest maximum and broadest range of water depth occurred on *M*. *spicatum*, *V*. *natans* and *V*. *natans*, respectively, in the monodominant communities (Fig 5). The minimum water depth of all the species was lower in the polydominant communities than that in the monodominant communities whereas the maximum and range of water depth of all the species were higher and broader in the polydominant communities than those in the monodominant communities (Fig 5, t, p<0.05).

### Frequency response

**Fig 6:**
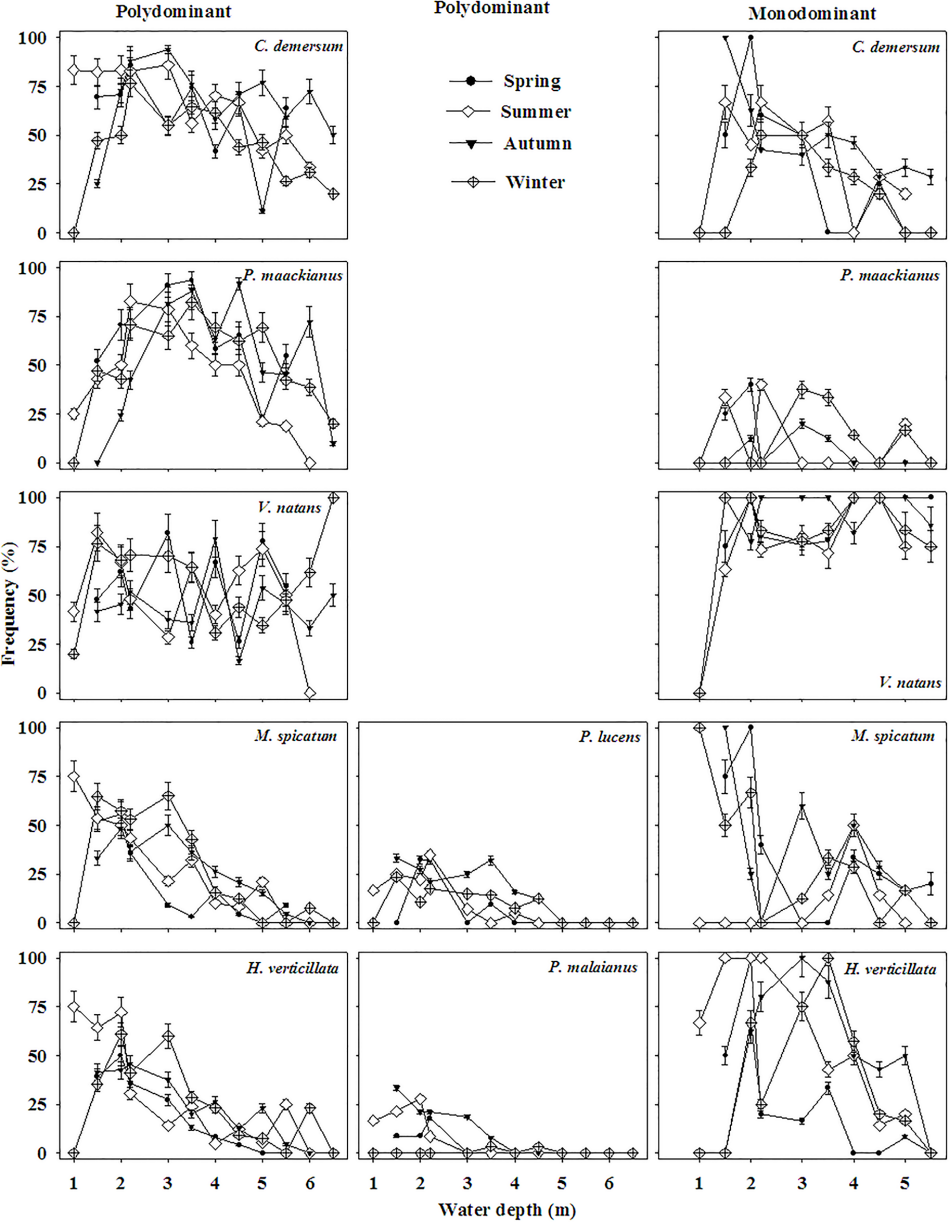
Seasonal frequency patterns of submersed species across the water depth gradient in the polydominant and monodominant communities.

In polydominant communities, frequency of *P*. *maackianus* showed a hump-back response patterns across the water depth gradient whereas frequency of *C*. *demersum* and *V*. *natans* maintained relative stable and frequency of the other species declined along the gradient (Fig 6). However, in monodominant communities, frequency of *V*. *natans* maintained relative stable with the water depth gradient whereas frequency of the other species declined with the gradient (Fig 6).

## Discussion

The field survey explicitly showed the hump-shaped correlations between community biomass, species richness and the water depth gradient both in the polydominant and monodominant communities. However, the distribution range of water depth, biomass and frequency of dominant species were different from those of companion species; furthermore, the distribution range of water depth, biomass and frequency of individual species were also different between polydominant and monodominant communities. We discussed the community and species responses to the water depth gradient in the following two parts, respectively.

### Community responses

We found significantly hump-shaped correlations between community biomass, species richness and the water depth, indicating that both species richness and productivity are the highest at an intermediate water depth in Lake Erhai [22–23]. In accordance with previous studies, the highest species richness was found at intermediate productivity along the water depth gradient both in the polydominant and monodominant communities [23–25]. The highest species richness and productivity were, however, at a deeper water depth in our study than in the study of Fu et al [7]. The reason may be that we only took submersed macrophytes into consideration and Fu et al [7] included floating macrophytes when analyzing the correlations.

### Species responses

Species responses to water depth gradient are more complicated than community responses. Dominant species, *Stuckenia pectinata*, *Najas marina*, *Ottelia acuminate* and *H*. *verticillata*, growing in low nutrients condition, are gradually replaced by the species growing in high nutrient level, such as *C*. *demersum*, *P*. *maackianus* and *V*. *natans* with the ongoing lake eutrophication as observed in our study [26–27], agreed with the increasing eutrophication indicated by the presence of *C*. *demersum* [28–29]. This replacement corresponds well with our observation that dominant species (except *P*. *maackianus* in polydominant communities) showed higher maximum, lower minimum and broader range of water depth than companion species and biomass and frequency of dominant species were greater than those of companion species (Fig 5).

Biomass and frequency of companion species (except *C*. *demersum* in monodominant communities) declined drastically with the water depth whereas those of dominant species demonstrated non-linear pattern across the water depth gradient. Our results are similar to the findings of Fu et al [7] when water depth is below 3 meters. Nevertheless, the actual biomass and frequency of submersed species along the water depth gradient can not be displayed in their study due to their limited study ranges of water depth in Lake Erhai [7]. Moreover, different responses to the water depth between dominant and companion species and the different responses in polydominant and monodominant communities can be discriminated in our study. These different responses between dominant and companion species may result from their different competitiveness for light in the current nutrient status in Lake Erhai [26–27]. The different responses between polydominant and monodominant communities are necessarily affected by the different number and / or kinds of dominant species [17]. In the shallow water, canopy former companion species can erect their most leaves above the water surface and grow at the highest position in the water column [7]. However, the potential growth to water surface for these species is restricted by their limited resources [30]. They may eventually suffer from low light stress with the increase of water depth since the amount of available light reduces distinctly along the water depth gradient [12]. Consequently, productivity and occurrence of companion species decreased sharply with the water depth.

For the three dominant species, light in the intermediate water depth may be relative sufficient because shading by floating and emergent plants and trees in the shallow water is very strong [11] and light transparence in the deep water is very low [12]. As shade-tolerant species, *V*. *natans* and *C*. *demersum* can outperform the erect species *P*. *maackianus* in both shallow and deep water because of their lower light compensation point of photosynthesis and lower C/N metabolic levels [10, 15]. However, in the intermediate water depth, *P*. *maackianus* competes out *V*. *natans* and *C*. *demersum* by producing longer shoots and more branches towards the water surface [14]. This unbalanced competitiveness among the three dominant species in the water depth gradient results in a higher biomass of *P*. *maackianus* in the intermediate water depth with relative high light and a lower biomass of *P*. *maackianus* in the shallow and deep water depth with relative low light [14]. Consequently, biomass percentage of *P*. *maackianus* showed a hump-back shaped pattern and biomass percentages of *V*. *natans* and *C*. *demersum* appeared U-shaped patterns across the water depth in the polydominant community (Fig 4). Nevertheless, lack of strong competitiveness from other submersed macrophytes may account for the increasing biomass percentage of *V*. *natans* with water depth in the monodominant communities (Fig 4). And the much higher biomass percentage of *P*. *maackianus* is owing to its mono-specific mats in many Chinese water bodies [31]. Similarly, biomasses of *P*. *maackianus* and *V*. *natans* showed hump-back patterns across the water depth gradient (Fig 3). However, the U-shape biomass pattern of *C*. *demersum* across the water depth in the polydominant community may due to its lower competitiveness in the intermediate water depth with relatively high light environment compared to other dominant species [7, 14–15].

For polydominant communities, hump-back shaped frequency of *P*. *maackianus* across the water depth agrees well with its higher competitive ability in relative high light condition and narrower distribution range of water depth whereas steady high frequencies of *V*. *natans* and *C*. *demersum* across the water depth are in accordance with their broader distribution range of water depth and shade-tolerant characters [7, 14–15, 32]. Based on the above findings, we can deduce that *P*. *maackianus* and *V*. *natans* prefer intermediate water depth and *C*. *demersum* prefer shallow and deep water in Lake Erhai because of habitat filtering and niche differentiation among aquatic plants [7]. Actually, in Lake Erhai, the optimum water depth ranges of *P*. *maackianus* and *C*. *demersum* in the polydominant communities are 2.5–4.5 m and 1–2 m or 5–6 m, respectively and that of *V*. *natans* is 3–5 m in the polydominant communities and 2.5–5 m in the monodominant communities. We firstly reported the optimum water depth ranges in the horizontal direction of three dominant submersed macrophytes in a natural freshwater lake.

Last but not least, only *V*. *natans* dominated in transects of S11-S13 with steep slope in the east lake. The reason might be that *V*. *natans* has deep and steady roots guaranteeing the root depth in the stiff clay [33]. *C*. *demersum* and *P*. *maackianus* dominated in transects of S1-S10 with gentle slope due to their rhizoid or shallow roots in the silt sediment [7, 34]. The lake basin morphology, force of wind-wave, animal herbivory and other factors may also contribute to the different distribution and assembly of submersed macrophytes in the polydominant and monodominant communities [2–5], which are out of the scope of the our study.

## Conclusion

In summary, species richness and community biomass showed hump-back shaped patterns along the water depth gradient in the polydominant and monodominant communities. Dominant species demonstrated broader distribution range of water depth than companion species. Frequency and biomass of companion species declined with the water depth whereas those of dominant species showed non-linear patterns across the water depth gradient. We can speculate that dominant *P*. *maackianus* and *V*. *natans* prefer intermediate water depth and *C*. *demersum* prefer shallow and deep water depth under the current nutrient status in Lake Erhai.

## Supporting information

S1 Table Recorded submersed species in the polydominant and monodominant communities (Symbol ‘‘+” and ‘‘-” indicates presence or absence, respectively). (DOCX)

S2 Table The regression coefficients and their corresponding 95% confidence intervals of the second-degree polynomial regressions between species richness and community biomass and the water depth of the three dominant submersed macrophytes in the polydominant and monodominant communities. (DOCX)
