# Supplementary material for: Optimum water depth ranges of dominant submersed macrophytes in a natural freshwater lake
Source: PLoS One. 2018 Mar 7;13(3):e0193176. doi: 10.1371/journal.pone.0193176 (PMC5841742; doi:10.1371/journal.pone.0193176)
Supplement: S1 Table — (DOCX) [file pone.0193176.s001.docx]

**S1 Table. Recorded submersed species in the polydominant and monodominant communities (Symbol ‘‘+’’ and ‘‘-’’ indicates presence or absence, respectively).**

| No | [specific](javascript:void(0);) | Family | genera | Polydominant | monodominant |
| --- | --- | --- | --- | --- | --- |
| 1 | *Potamogeton praelongus* | Potamogetonaceae | Potamogeton | + | - |
| 2 | *Potamogeton crispus* | Potamogetonaceae | Potamogeton | + | + |
| 3 | *Potamogeton distinctus* | Potamogetonaceae | Potamogeton | + | - |
| 4 | *Potamogeton intortifolius* | Potamogetonaceae | Potamogeton | + | + |
| 5 | *Potamogeton lucens* | Potamogetonaceae | Potamogeton | + | + |
| 6 | *Potamogeton maackianus* | Potamogetonaceae | Potamogeton | + | + |
| 7 | *Potamogeton malaianus* | Potamogetonaceae | Potamogeton | + | + |
| 8 | *Stuckenia pectinata* | Potamogetonaceae | Potamogeton | + | + |
| 9 | *Potamogeton perfoliatus* | Potamogetonaceae | Potamogeton | + | + |
| 10 | *Potamogeton pusillus* | Potamogetonaceae | Potamogeton | + | - |
| 11 | *Najas marina* | Najadaceae | Najas | + | - |
| 12 | *Hydrilla verticillata* | Hydrocharitaceae | Hydrilla | + | + |
| 13 | *Ottelia acuminata* | Hydrocharitaceae | Ottelia | + | - |
| 14 | *Vallisneria natans* | Hydrocharitaceae | Vallisneria | + | + |
| 15 | *Ceratophyllum demersum* | Ceratophyllaceae | Ceratophyllum | + | + |
| 16 | *Myriophyllum spicatum* | Haloragidaceae | Myriophyllum | + | + |
| 17 | *Utricularia aurea* | Lentibulariaceae | Utricularia | + | + |
| 18 | *Chara* SPP. | Characeae | Chara | + | + |
