# Supplementary material for: Optimum water depth ranges of dominant submersed macrophytes in a natural freshwater lake
Source: PLoS One. 2018 Mar 7;13(3):e0193176. doi: 10.1371/journal.pone.0193176 (PMC5841742; doi:10.1371/journal.pone.0193176)
Supplement: S2 Table — (DOCX) [file pone.0193176.s002.docx]

Appendix Table 3 The regression coefficients and their corresponding 95% confidence intervals of the second-degree polynomial regressions between species richness and community biomass and the water depth of the three dominant submersed macrophytes in the polydominant and monodominant communities

| Community | Polycommunity | | Monocommunity | |
| --- | --- | --- | --- | --- |
|  | Species richness | Community biomass | Species richness | Community biomass |
| β_0_ | -2.578 | -14.200 | -3.051 | -13.168 |
| β_0_ 95% confidence interval | -4.253 - -0.903 | -15.800 - -12.600 | -4.131 - -1.971 | -15.789 - -10.547 |
| Β_1_ | 6.377 | 15.599 | 4.239 | 13.008 |
| Β_1_ 95% confidence interval | 5.432 – 7.322 | 14.697 – 16.501 | 3.591 – 4.887 | 11.435 – 14.581 |
| Β_2_ | -0.876 | -1.982 | -0.569 | -1.693 |
| Β_2_ 95% confidence interval | -0.992 - -0.760 | -2.092 - -1.872 | -0.654 - -0.484 | -1.899 - -1.478 |
